# Avian oncogenic herpesvirus antagonizes the cGAS-STING DNA-sensing pathway to mediate immune evasion

**DOI:** 10.1371/journal.ppat.1007999

**Published:** 2019-09-20

**Authors:** Kai Li, Yongzhen Liu, Zengkun Xu, Yu Zhang, Dan Luo, Yulong Gao, Yingjuan Qian, Chenyi Bao, Changjun Liu, Yanping Zhang, Xiaole Qi, Hongyu Cui, Yongqiang Wang, Li Gao, Xiaomei Wang

**Affiliations:** 1 Avian Immunosuppressive Diseases Division, State Key Laboratory of Veterinary Biotechnology, Harbin Veterinary Research Institute, Chinese Academy of Agricultural Sciences, Harbin, China; 2 MOE Joint International Research Laboratory of Animal Health and Food Safety, College of Veterinary Medicine, Nanjing Agricultural University, Nanjing, China; University of Southern California, UNITED STATES

## Abstract

The cellular DNA sensor cGMP-AMP synthase (cGAS) detects cytosolic viral DNA via the stimulator of interferon genes (STING) to initiate innate antiviral response. Herpesviruses are known to target key immune signaling pathways to persist in an immune-competent host. Marek’s disease virus (MDV), a highly pathogenic and oncogenic herpesvirus of chickens, can antagonize host innate immune responses to achieve persistent infection. With a functional screen, we identified five MDV proteins that blocked beta interferon (IFN-β) induction downstream of the cGAS-STING pathway. Specifically, the MDV major oncoprotein Meq impeded the recruitment of TANK-binding kinase 1 and IFN regulatory factor 7 (IRF7) to the STING complex, thereby inhibiting IRF7 activation and IFN-β induction. Meq overexpression markedly reduced antiviral responses stimulated by cytosolic DNA, whereas knockdown of Meq heightened MDV-triggered induction of IFN-β and downstream antiviral genes. Moreover, Meq-deficient MDV induced more IFN-β production than wild-type MDV. Meq-deficient MDV also triggered a more robust CD8+ T cell response than wild-type MDV. As such, the Meq-deficient MDV was highly attenuated in replication and lymphoma induction compared to wild-type MDV. Taken together, these results revealed that MDV evades the cGAS-STING DNA sensing pathway, which underpins the efficient replication and oncogenesis. These findings improve our understanding of the virus-host interaction in MDV-induced lymphoma and may contribute to the development of novel vaccines against MDV infection.

## Author summary

Marek’s disease virus (MDV) is an avian oncogenic herpesvirus that causes a fatal disease in poultry worldwide. Chickens infected with MDV become more susceptible to secondary viral or bacterial infections. However, the mechanisms of MDV-induced immunosuppression and tumorigenesis remain largely unknown. The cGAS-STING pathway is crucial for innate immune responses against both microbial pathogens and intrinsic tumors. Here we identified the MDV oncoprotein, Meq, as an inhibitor of the cGAS-STING DNA-sensing pathway. Mechanistically, Meq interacted with STING and IRF7, and impaired the recruitment of TBK1 and IRF7 to the STING complex, thus inhibiting IRF7 activation and IFN-β induction. Loss of Meq potently enhanced innate immune response, while impaired the replication and oncogenesis of MDV in chickens. Our findings reveal an important mechanism of immune evasion of MDV, instructing us on the virus-host interaction in MDV-induced lymphoma and potential new means to develop MDV vaccine.

## Introduction

Herpesviruses are important pathogens associated with a wide range of diseases in humans and animals. In particular, Marek’s disease virus (MDV) constitutes a highly pathogenic and oncogenic herpesvirus of chickens [1]. As a disease that affects poultry worldwide with economic implications, Marek’s disease (MD) has contributed substantially to our understanding of herpesvirus-associated oncogenicity [2]. MD lymphomas exhibit many biological parallels with the lymphoid neoplasias associated with human herpesviruses such as Epstein-Barr virus (EBV) [3]. Despite the success of vaccination in controlling MD over the last 40 years, continuous evolution of virulence among MDV strains remains a major challenge for sustainable control of this disease [4]. A better understanding of MDV-host interactions is, therefore, important to not only elucidate the events in oncogenesis but also develop more effective vaccines to combat infection.

Evasion of the host innate immune response is essential for herpesviruses to successfully establish infection, latency, and lifelong persistence in the host [5]. Innate responses are initiated upon the detection of invading pathogens by various host pattern-recognition receptors that recognize conserved pathogen-associated molecular patterns and trigger the production of type I interferons (IFNs) and other antiviral factors [6, 7]. In addition to Toll-like receptors, retinoic acid-inducible gene I-like receptors, and Nod-like receptors, several cytosolic DNA sensors have been recently discovered [8, 9]. Among these DNA sensors, cyclic GMP-AMP (cGAMP) synthase (cGAS) is currently considered the principal sensor of cytosolic DNA in different cell types [10, 11]. Upon binding DNA, cGAS utilizes GTP and ATP to produce cGAMP, the latter of which activates the downstream adaptor protein stimulator of interferon genes (STING), which then recruits TANK-binding kinase 1 (TBK1) to phosphorylate and activate IFN regulatory factor 3 (IRF3) and IRF7, ultimately leading to IFN-β production [9, 12]. STING also activates nuclear factor (NF)-κB, which functions together with IRF3/IRF7 to initiate transcription of IFNs and inflammatory cytokines [9, 12]. Recently, the cGAS-STING DNA-sensing pathway was reported to play an important role in type I IFN responses against herpesviruses including herpes simplex virus 1 (HSV-1), Kaposi sarcoma herpesvirus (KSHV), and human cytomegalovirus (HCMV) [13–15]. Moreover, a number of viral proteins that inhibit type I IFN production through modulation of this signaling pathway have been identified such as HSV-1 UL41 [16], KSHV vIRF1 [14], and HCMV UL31 [17].

The oncogenic MDV encodes a basic leucine zipper (bZIP) protein, Meq, which is consistently expressed in all tumor and latently infected cells and has been suggested to represent the major oncoprotein of MDV [18, 19]. Earlier studies showed that expression of Meq alone was sufficient to induce transformation in rat cells [20]. A direct role of Meq in tumorigenesis has been demonstrated using a Meq-null mutant virus that failed to induce tumors in chickens [18]. As a bZIP protein with characteristics similar to those of oncoproteins such as v-Jun, Meq is able to dimerize with itself along with other ZIP proteins such as c-Jun, c-Fos, and ATF-3 [21, 22]. In addition, Meq has non-bZIP interactions with transcriptional corepressor C-terminal-binding protein and tumor suppressor protein p53, which was shown to be essential for the oncogenic properties of Meq [23, 24]. Meq can also inhibit apoptosis through the regulation of Bcl2 and p53 [24–26]. However, despite these observations, the molecular mechanisms of Meq-induced lymphoma are not completely understood.

In this study, we aimed to identify MDV proteins that inhibit the cGAS-STING pathway and elucidate how this inhibition is related to lymphoma development in chickens. We found that MDV oncoprotein Meq acted as an important inhibitor of the cGAS-STING DNA-sensing pathway. Mechanistically, Meq bound to STING and IRF7, and subsequently impaired assembly of the STING-TBK1-IRF7 complex, thereby efficiently inhibiting the induction of type I IFNs and downstream antiviral genes upon MDV infection or cytosolic DNA stimulation. Our findings reveal a novel strategy through which MDV evades host innate immunity and provide insight into the mechanisms by which MDV establishes latency and transformation.

## Results

### MDV inhibits IFN-β induction during the late phase of viral infection

**Fig 1:**
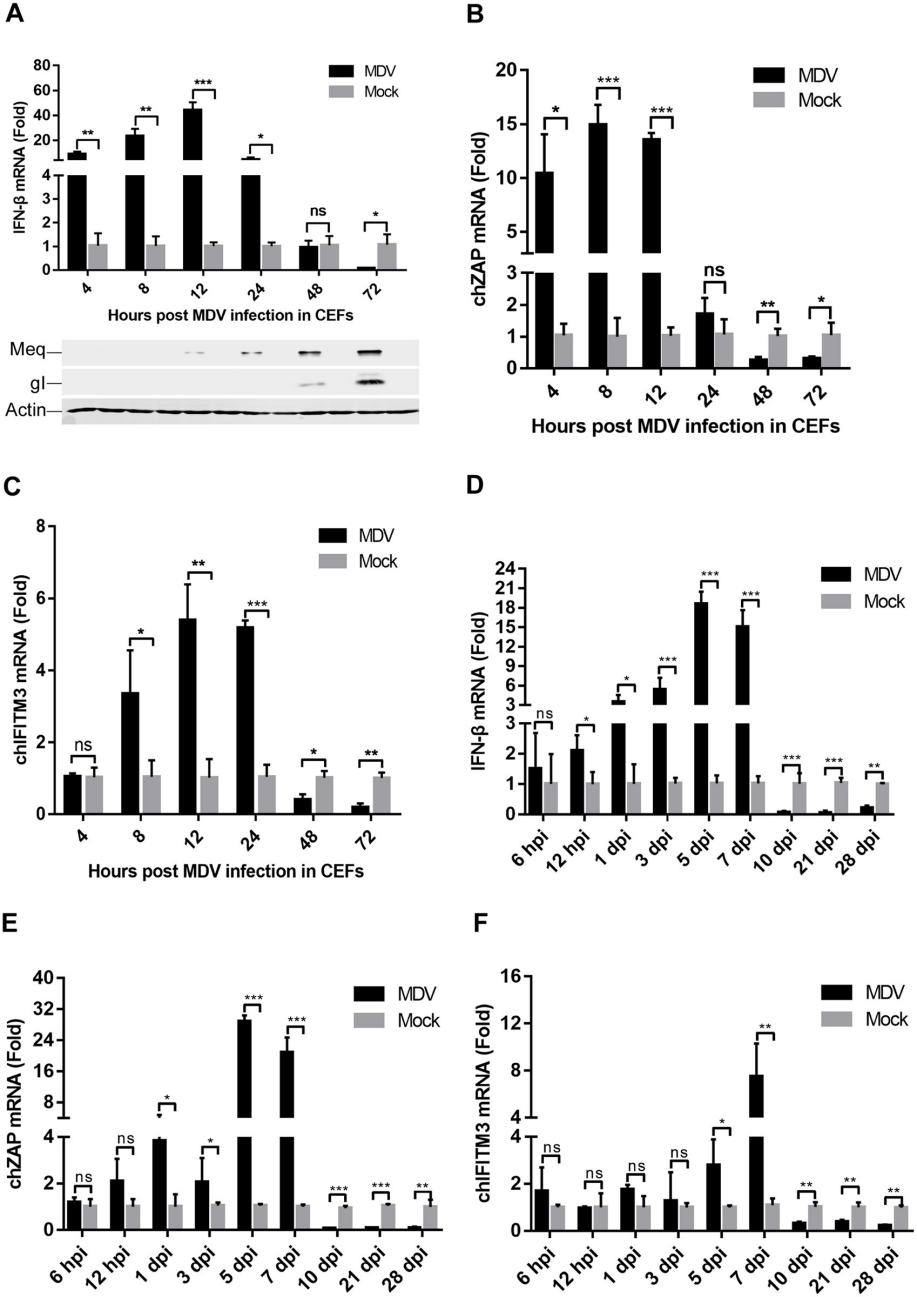
MDV suppresses the induction of IFN-β and downstream antiviral genes during the late phase of viral infection. (A-C) CEFs were infected with the virulent MDV GA strain at a multiplicity of infection (MOI) of 0.1. The mRNA levels of *IFN-β* (A) and IFN-stimulated genes (ISGs) chicken ZAP (ch*ZAP*) (B) and chicken IFN-inducible transmembrane protein 3 (ch*IFITM3*) (C) were measured by real-time qPCR from 4 to 72 hpi. The expression of MDV protein Meq and gI during viral infection was monitored by western blotting. (D-F) One-day-old specific pathogen-free chickens were inoculated subcutaneously with 2000 PFUs of MDV GA virus, and the mRNA levels of *IFN-β* (D) and chicken ISGs *ZAP* (E) and *IFITM3* (F) in the spleen samples were measured by real-time qPCR. The relative amounts of *IFN-β*, *ZAP*, and *IFITM3* mRNA were normalized to the *actin* mRNA level in each sample, and the fold differences were compared with those in the mock samples. *: p < 0.05, **: p < 0.01, ***: p < 0.001; ns: no significant difference.

MDV infection causes immunosuppression and lymphoma in chickens [27, 28]; therefore, it is highly plausible that MDV inhibits type I IFN induction and escapes the host innate immunity during viral infection. To test this idea, we infected chicken embryo fibroblasts (CEFs) with the virulent MDV GA strain and analyzed mRNA expression of IFN-β by real-time quantitative polymerase chain reaction (qPCR). As shown in Fig 1A, IFN-β induction in CEFs upon MDV infection was prominent at early time points (4 to 12 h) but decreased at later time points (24 to 72 h) postinfection (pi). Furthermore, MDV infection also inhibited transcription of the chicken IFN-stimulated genes (ISGs) *ZAP* and IFN-inducible transmembrane protein 3 (*IFITM3*) at 48 and 72 hpi in CEFs (Fig 1B and 1C). Consistent with the inhibition of IFN-β induction, various MDV proteins were expressed during the late phase of viral infection (Fig 1A), suggesting that these viral proteins might contribute to the modulation of the IFN-β response during viral infection.

We also infected chickens with the oncogenic MDV GA strain and assessed IFN-β induction. Indeed, MDV infection triggered an IFN-β response during the early cytolytic phase (within 12 hpi to 7 dpi). Remarkable, IFN-β induction was significantly decreased in MDV-infected chickens during the reactivation and transformation phases (within 10 to 28 dpi) (Fig 1D). Consistent with this, the transcription of *ZAP* and *IFITM3* was also greatly reduced at the later time points of MDV infection *in vivo* (Fig 1E and 1F). These results conclude that MDV inhibits host innate immune responses during the late phase of viral infection, which may contribute to the reactivation and neoplastic transformation of MDV in chickens.

### Multiple MDV proteins block cGAS-STING-mediated IFN-β activation

**Fig 2:**
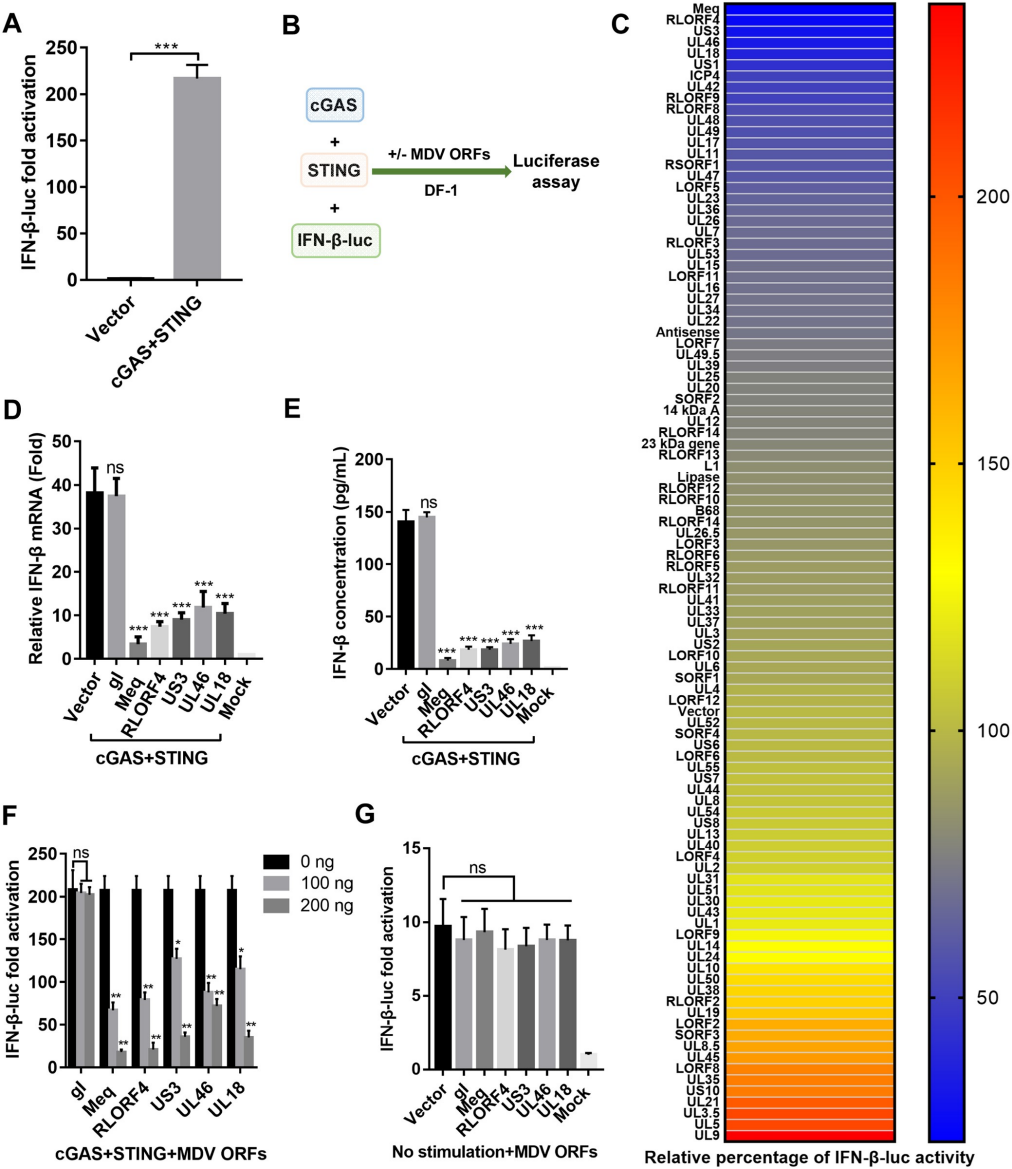
Screening of MDV open reading frames (ORFs) that modulate the cGAS-STING pathway. (A) DF-1 cells were cotransfected with *IFN-β* promoter luciferase reporter and various plasmids (pCAGGS or pCAGGS-cGAS-HA and pCAGGS-STING-HA combined). The luciferase activity was measured at 36 h posttransfection. (B) Schematic of the screening assay. DF-1 cells were transfected with the same amount of cGAS and STING expression plasmid, plus each of MDV ORF expression plasmid or the empty vector. (C) Heat map of the effects of MDV ORFs on the cGAS-STING pathway. Higher *IFN-β* promoter luciferase activation levels are indicated by red, whereas lower levels are indicated by blue, which corresponds to a higher degree of inhibition. (D) The top five MDV ORF inhibitors and the MDV gI ORF were cotransfected with cGAS and STING expression plasmids into DF-1 cells. At 36 h posttransfection, *IFN-β* mRNA levels were measured by real-time qPCR. The relative amount of *IFN-β* mRNA was normalized to the *actin* mRNA level in each sample, and the fold changes were compared with those in the mock controls. (E) The top five MDV ORF inhibitors and the gI ORF were cotransfected with cGAS and STING expression plasmids into DF-1 cells, and IFN-β protein levels were measured by enzyme-linked immunosorbent assay 36 h posttransfection. (F) Varying doses of the top five MDV ORF inhibitors and the gI ORF were cotransfected with cGAS and STING expression plasmids, and *IFN-β* promoter luciferase activity was measured at 36 h posttransfection. (G) The top five MDV ORF inhibitors and the gI ORF were transfected into DF-1 cells, and *IFN-β* promoter luciferase activity was measured at 36 h posttransfection. *: p < 0.05, **: p < 0.01, ***: p < 0.001; ns: no significant difference.

Because the cGAS-STING pathway plays a critical role in the induction of type I IFNs in response to herpesviruses [13–15], the inhibition of IFN-β induction during MDV infection suggests that MDV may encode proteins that antagonize this pathway. DF-1, a chicken fibroblast cell line, is known to respond to foreign DNA such as IFN stimulatory DNA (ISD) and poly(dA:dT) [29]. In DF-1 cells, the *IFN-β* promoter was highly activated when the same amounts of cGAS and STING expression plasmids were cotransfected together with an *IFN-β* promoter luciferase reporter construct (Fig 2A). Using this assay, we screened for viral proteins that could inhibit the activation of the *IFN-β* promoter induced by cGAS and STING (Fig 2B). This screen identified several MDV proteins, including Meq, RLORF4, US3, UL46, and UL18 that could reduce IFN-β induction by 3- to 5-fold (Fig 2C). The inhibitory effect of these MDV proteins was validated by measuring the *IFN-β* mRNA levels using qPCR (Fig 2D) as well as IFN-β protein levels using enzyme-linked immunosorbent assay (ELISA) (Fig 2E) in DF-1 cells. We further found that each viral inhibitor inhibited activation of the *IFN-β* promoter in a dose-dependent manner (Fig 2F), whereas another MDV protein, gI, showed no effect on IFN-β induction induced by cGAS and STING. These clones did not affect the basal *IFN-β* promoter activity in the absence of exogenous cGAS and STING expression, indicating the specificity of these MDV proteins on the cGAS-STING pathway (Fig 2G). Additionally, the five candidates exhibited different effects on the activation of the IFN-β promoter induced by TBK1 and IRF7 (S1 Fig), suggesting that these viral proteins may be able to inhibit this pathway at multiple nodes.

### MDV Meq suppresses IFN-β induction in response to viral DNA

**Fig 3:**
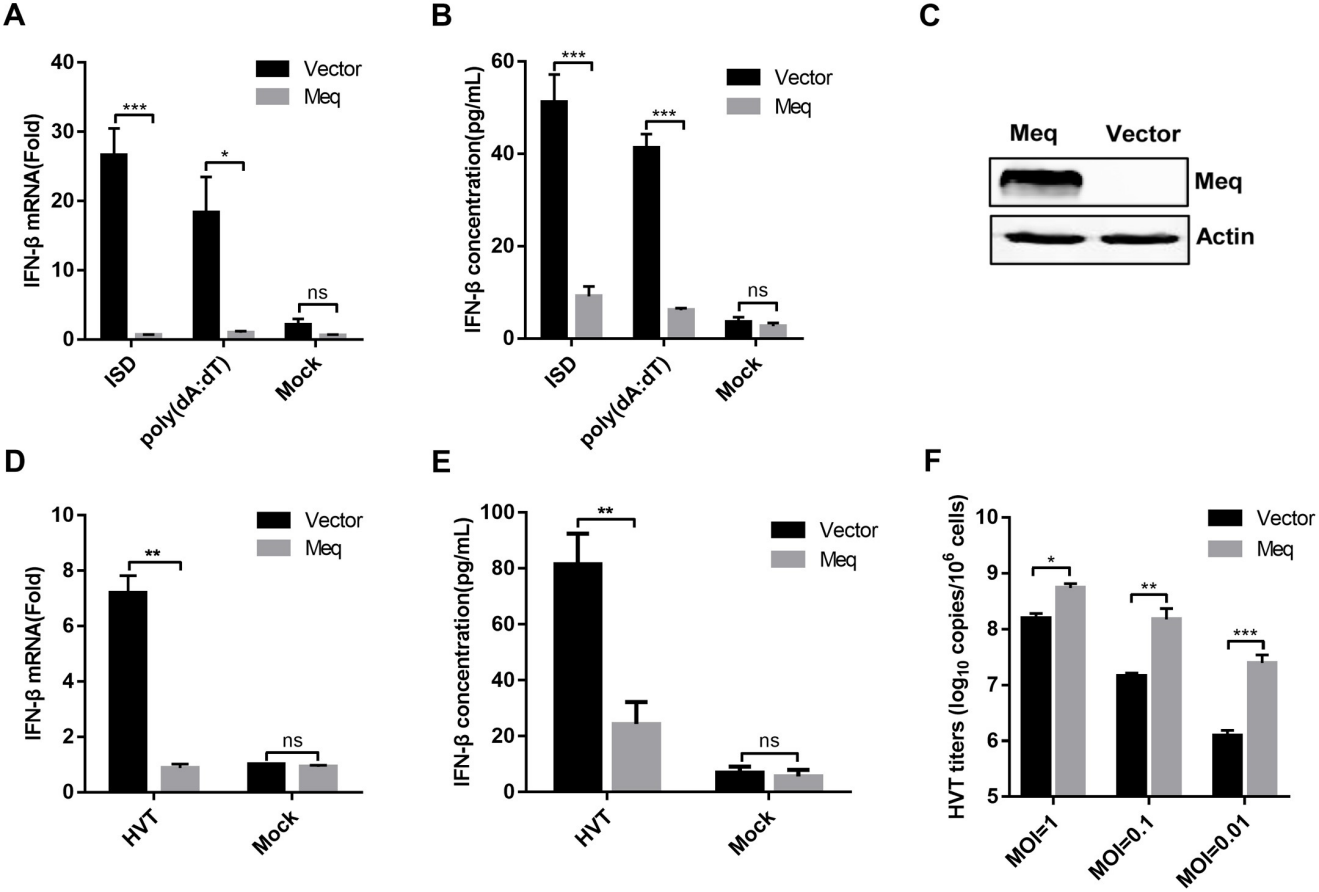
MDV Meq suppresses IFN-β induction in response to viral DNA and promotes viral replication. (A, B) DF-1 cells were transfected with empty vector or Meq expression plasmid. After 24 h, they were transfected with IFN stimulatory DNA (ISD) fragments or poly(dA:dT). *IFN-β* mRNA was measured by real-time qPCR 8 h post ISD or poly(dA:dT) transfection (A), and IFN-β protein levels were measured by enzyme-linked immunosorbent assay (ELISA) 24 h post ISD or poly(dA:dT) transfection (B). (C) The expression of Meq in DF-1 cells transduced with empty vector or Meq-expressing lentivirus was monitored by western blotting. (D, E) DF-1 cells transduced with empty vector or Meq-expressing lentivirus were left uninfected or infected with HVT (multiplicity of infection (MOI) = 0.1). *IFN-β* mRNA in these cells was measured by real-time qPCR 12 hpi (D), and IFN-β protein was measured by ELISA 24 hpi (E). (F) Transduced DF-1 cells were infected with varying doses of HVT (MOI = 1, 0.1, or 0.01). At 48 hpi, the HVT viral titer was tested with real-time qPCR. The relative level of *IFN-β* mRNA was normalized to *actin* in each sample, and the fold differences between the treated samples and the mock samples were calculated. *: p < 0.05, **: p < 0.01, ***: p < 0.001; ns: no significant difference.

In this study, we focused on the MDV protein Meq, because it showed the strongest ability to inhibit the cGAS-STING pathway. Moreover, Meq is unique to MDV and considered the major viral oncoprotein [19]. To confirm the inhibitory effect of Meq on the DNA-sensing pathway, we transfected DF-1 cells with a Meq expression plasmid, and stimulated cells with ISD or poly(dA:dT) at 24 h later. As shown in Fig 3A and 3B, Meq markedly inhibited IFN-β induction triggered by the transfected DNA mimics in DF-1 cells at both the mRNA and protein levels. We next generated stable DF-1 cells ectopically expressing Meq via lentiviral-mediated transduction (Fig 3C) and tested whether Meq can suppress IFN-β induction provoked by DNA virus infection. We infected the empty vector- and Meq-expressing cells with herpesvirus of turkey (HVT) and found that Meq expression reduced IFN-β induction against HVT, compared with that of the control, at both the transcriptional and protein levels (Fig 3D and 3E). Consistently, different multiplicities of infection (MOI) of HVT (1, 0.1, and 0.01) replicated to higher titers in the Meq-expressing cells compared with those in the vector control cells (Fig 3F). These results indicate that Meq inhibits IFN-β induction to promote viral replication.

### Meq deficiency enhances MDV-triggered IFN-β induction

**Fig 4:**
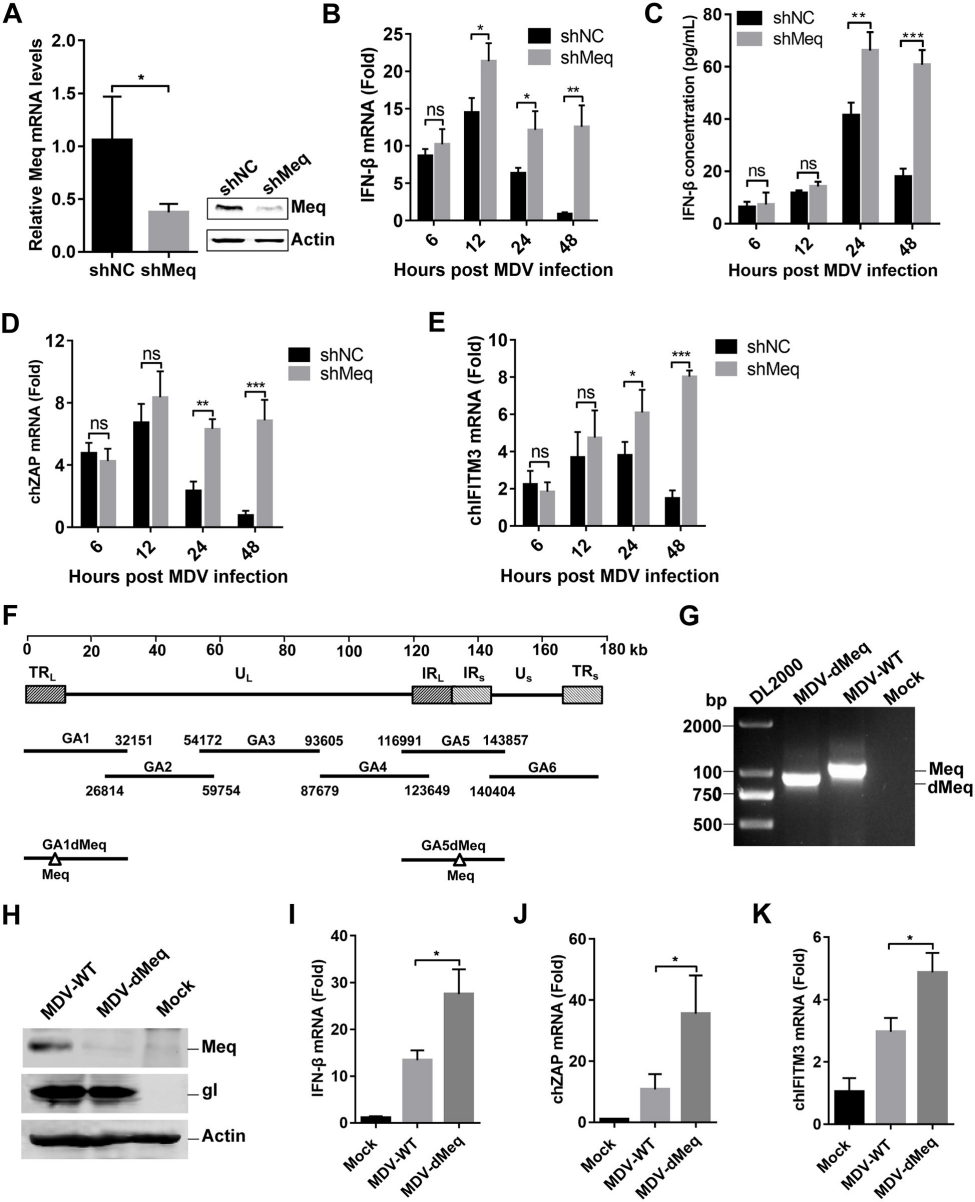
Meq deficiency enhances MDV-triggered induction of *IFN-β* and downstream antiviral genes. (A) Real-time qPCR and western blot analysis of the CEFs lentivirally transduced with Meq-specific small hairpin RNAs (shMeq) or a control shRNA (shNC) after 48 h of MDV infection. (B, C) CEFs transduced with shMeq or shNC were infected with MDV (multiplicity of infection (MOI) = 0.1) for the indicated times. *IFN-β* mRNA levels were measured by real-time qPCR (B), and IFN-β protein levels were measured by enzyme-linked immunosorbent assay (C). (D, E) CEFs transduced with shNC or shMeq were infected with MDV (MOI = 0.1) for the indicated times. The mRNA levels of chicken ZAP (ch*ZAP*) (D) and chicken IFN-inducible transmembrane protein 3 (ch*IFITM3*) (E) were measured by real-time qPCR. (F) Schematic diagram of the recombinant fosmids for constructing the wild-type MDV (MDV-WT) and the Meq-deficient MDV (MDV-dMeq) viruses. (G) PCR analyses of the Meq-deficient MDV. (H) Western blot analysis of the CEFs infected with MDV-WT or MDV-dMeq using the indicated antibodies. (I-K) Effects of Meq deficiency on transcription of *IFN-β* and downstream antiviral genes *in vitro*. CEFs were infected with MDV-WT or MDV-dMeq (multiplicity of infection (MOI) = 0.1) for 12 h prior to analysis of *IFN-β* (I), ch*ZAP* (J), and ch*IFITM3* (K) mRNA levels. The amounts of *IFN-β*, ch*ZAP*, or ch*IFITM3* mRNA were normalized to the *actin* mRNA level in each sample, and the fold difference relative to the mock controls at each time point was determined. *: p < 0.05, **: p < 0.01, ***: p < 0.001; ns: no significant difference.

To investigate the roles of endogenous Meq in the antiviral response to MDV, we generated CEFs stably expressing Meq-specific small hairpin RNAs (shRNAs) or a control shRNA. The knockdown of Meq expression was confirmed by qPCR and western blotting at the transcriptional and protein levels during MDV infection, respectively (Fig 4A). As shown in Fig 4B and 4C, Meq knockdown promoted IFN-β transcription and protein secretion in response to MDV infection at 24 and 48 hpi. Moreover, transcription of chicken ISGs *ZAP* and *IFITM3* induced by MDV infection was markedly increased in Meq-knockdown cells compared with that in cells transduced with control shRNA (Fig 4D and 4E).

We further generated Meq-deficient MDV (MDV-dMeq) using overlapping fosmid clones of the virulent MDV strain GA (Fig 4F). Deletion of the *Meq* gene from the MDV genome was confirmed by PCR analyses (Fig 4G). As expected, wild-type MDV (MDV-WT) expressed both viral proteins gI and Meq, whereas MDV-dMeq expressed gI but not Meq (Fig 4H). We next examined the ability of MDV-WT and MDV-dMeq to induce *IFN-β* and downstream antiviral genes. The results indicated that MDV-dMeq induced significantly higher mRNA levels of *IFN-β*, *ZAP*, and *IFITM3* than MDV-WT in CEFs (Fig 4I–4K). Collectively, these results demonstrate that Meq deficiency increases IFN-β induction during MDV infection.

### Meq interacts with STING and IRF7

**Fig 5:**
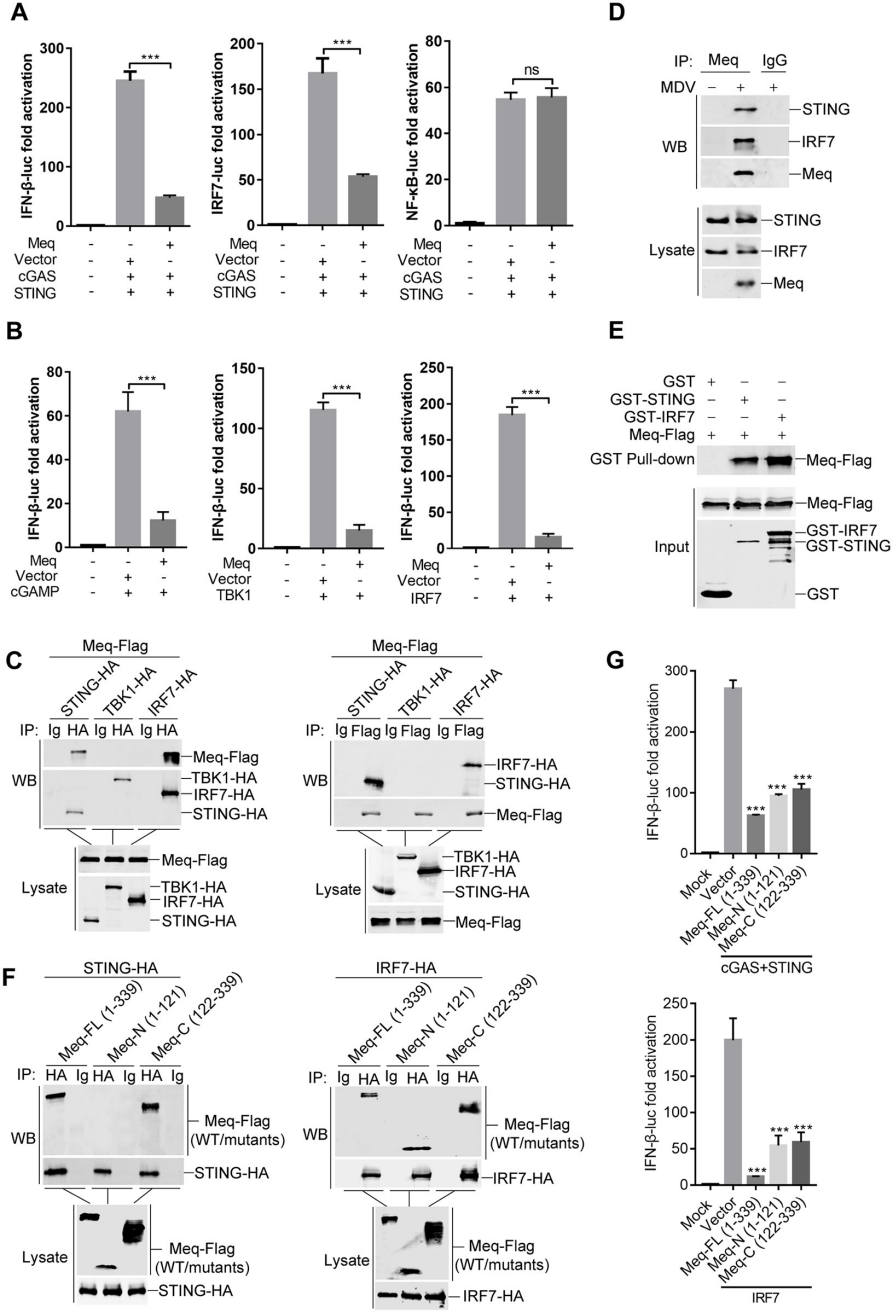
Meq interacts with STING and IRF7. (A) The IFN-β-luc, IRF7-luc, or NF-κB-luc reporter was cotransfected with cGAS and STING constructs as well as Meq-Flag plasmid or empty vector into DF-1 cells. After 36 h, cells were harvested and analyzed using the dual-luciferase reporter assay. (B) DF-1 cells were transfected with cGAMP or plasmid expressing TBK1 or IRF7, together with IFN-β-luc reporter and Meq-Flag plasmid or an empty vector. The dual-luciferase reporter assay was performed 36 h posttransfection, and the fold relative to the mock controls was determined. (C) HEK293T cells were transfected with the indicated plasmids for 36 h before coimmunoprecipitation and immunoblot analysis with the indicated antibodies. (D) Coimmunoprecipitation and immunoblot analyses were performed with the endogenous proteins from the CEFs left uninfected or infected with MDV. (E) Purified GST, GST-STING, or GST-IRF7 were used to pull down transiently expressed Meq-Flag as indicated. (F) Full length Meq (Meq-FL), and N- (Meq-N) or C-terminally truncated forms of Meq (Meq-C) were transfected together with STING-HA or IRF7-HA plasmids into HEK293T cells for 36 h before coimmunoprecipitation and immunoblot analysis with the indicated antibodies. (G) DF-1 cells were transfected with IFN-β-luc reporter, and expression plasmids for cGAS, STING, IRF7, Meq, and its truncation mutants for 36 h before luciferase assays. ***: p < 0.001; ns: no significant difference.

Chickens are IRF3-deficient, and the transcription of IFN-β in chickens is dependent on the binding of IRF7 and NF-κB transcription factors to distinct regulatory domains in the IFN-β promoter [30, 31]. To delineate the mechanism of IFN-β inhibition by Meq, we first measured the effects of Meq on IRF7 and NF-κB activation using a dual-luciferase reporter assay [29]. As shown in Fig 5A, Meq suppressed cGAS-STING-induced expression of IFN-β- and IRF7-dependent reporter genes, but did not significantly alter the NF-κB-dependent luciferase activity, suggesting that Meq inhibits the activation of IRF7 but not that of NF-κB. Reporter assays further indicated that Meq could inhibit IFN-β activation induced by cGAMP, and the downstream components TBK1 and IRF7 (Fig 5B), which suggest that Meq may target multiple steps of the cGAS-STING pathway. We then performed coimmunoprecipitation and found that Meq was associated with STING and IRF7, but not TBK1 (Fig 5C). Coimmunoprecipitation experiments using endogenous proteins indicated that Meq was associated with STING and IRF7 in MDV-infected CEFs (Fig 5D). *In vitro* glutathione S-transferase (GST)-pull down assays further confirmed that Meq interacted directly with STING and IRF7 (Fig 5E). We further mapped the interaction domains of Meq, and found that the C-terminal transactivation domain of Meq (aa 122–339) interacted with STING, while both the N-terminal bZIP domain (aa 1–121) and the C-terminal domain of Meq (aa 122–339) interacted with IRF7 (Fig 5F). Additionally, both the N-terminal and the C-terminal domains of Meq inhibited the activation of IFN-β promoter mediated by cGAS-STING or IRF7 (Fig 5G). These results collectively support the conclusion that Meq inhibits the innate antiviral response by targeting STING and IRF7.

### Meq impairs the recruitment of TBK1 and IRF7 to the STING adaptor

**Fig 6:**
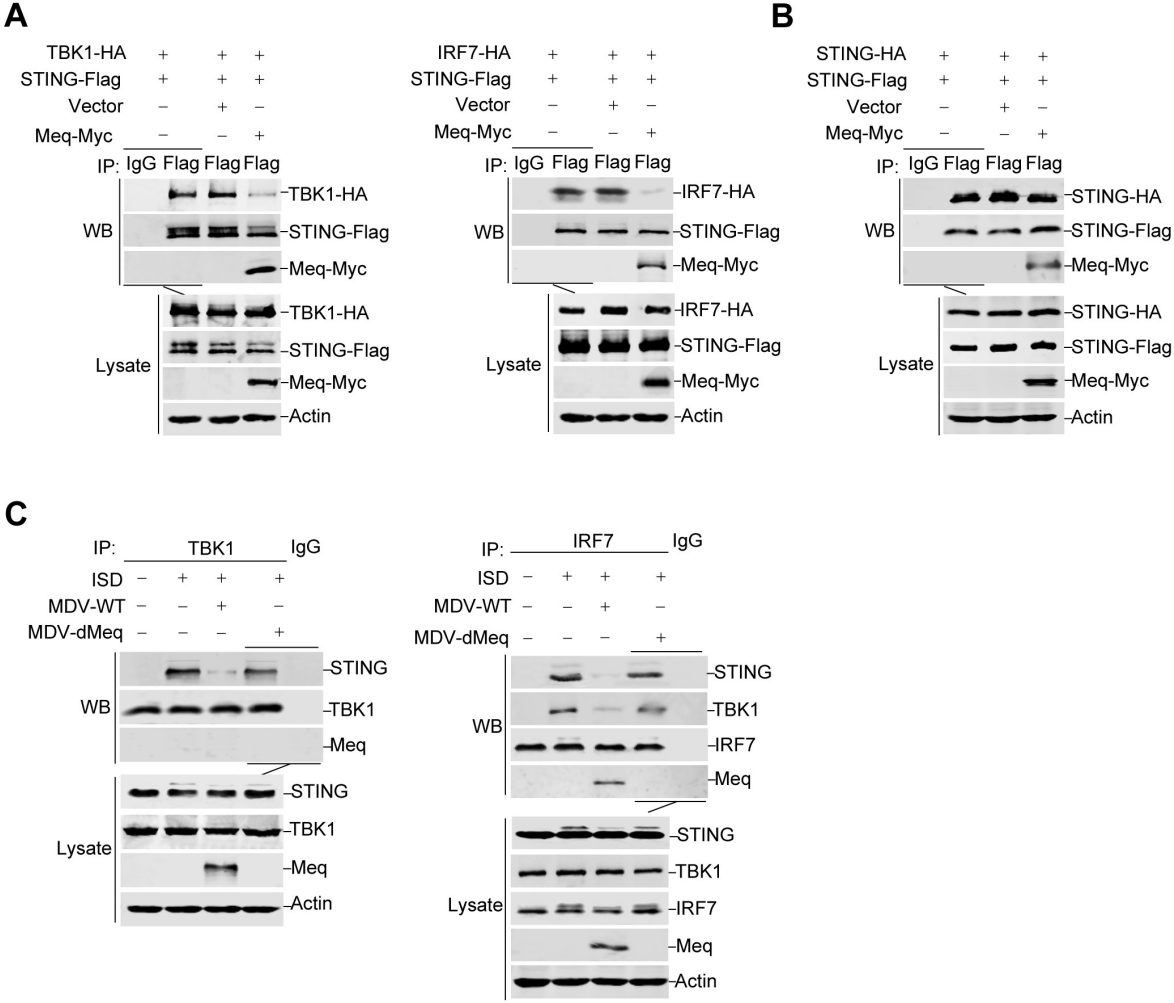
Meq impairs the recruitment of TBK1 and IRF7 to STING adaptor. (A) DF-1 cells were transfected with the indicated plasmids for 36 h before coimmunoprecipitation and immunoblot analysis with the indicated antibodies. (B) The STING-HA plasmid was cotransfected with STING-Flag with or without Meq-Myc into DF-1 cells. After 36 h of transfection, coimmunoprecipitation and immunoblot were performed with the indicated antibodies. (C) The CEFs were first mock infected or infected with wild-type MDV (MDV-WT) or Meq-deficient MDV (MDV-dMeq) and then transfected with ISD for another 12 h. The cells were lysed and subjected to immunoprecipitation assays with the indicated antibodies.

It was previously shown that upon DNA stimulation, STING recruits both TBK1 and IRF7 to form the STING signalosome that enables IRF7 phosphorylation by TBK1, thus activating the IFN-β induction [9, 12]. Here we found that chicken STING was associated with TBK1 and IRF7 in coimmunoprecipitation assays; whereas Meq inhibited the association of STING with TBK1 or IRF7 (Fig 6A), but not the dimerization of STING (Fig 6B). Coimmunoprecipitation assays with endogenous proteins further indicated that the amount of TBK1 and IRF7 recruited to the STING complex was decreased in CEFs infected with wild-type MDV, but not the Meq-deficient MDV (Fig 6C). Similarly, the association of TBK1 with IRF7 was also inhibited in CEFs infected with wild-type MDV (Fig 6C). These results show that Meq impairs the assembly of the STING-TBK1-IRF7 complex. In comparison, the interaction of STING with the IκB kinase β (IKKβ) and melanoma differentiation-associated gene 5 (MDA5) was not affected by Meq overexpression (S2 Fig).

**Fig 7:**
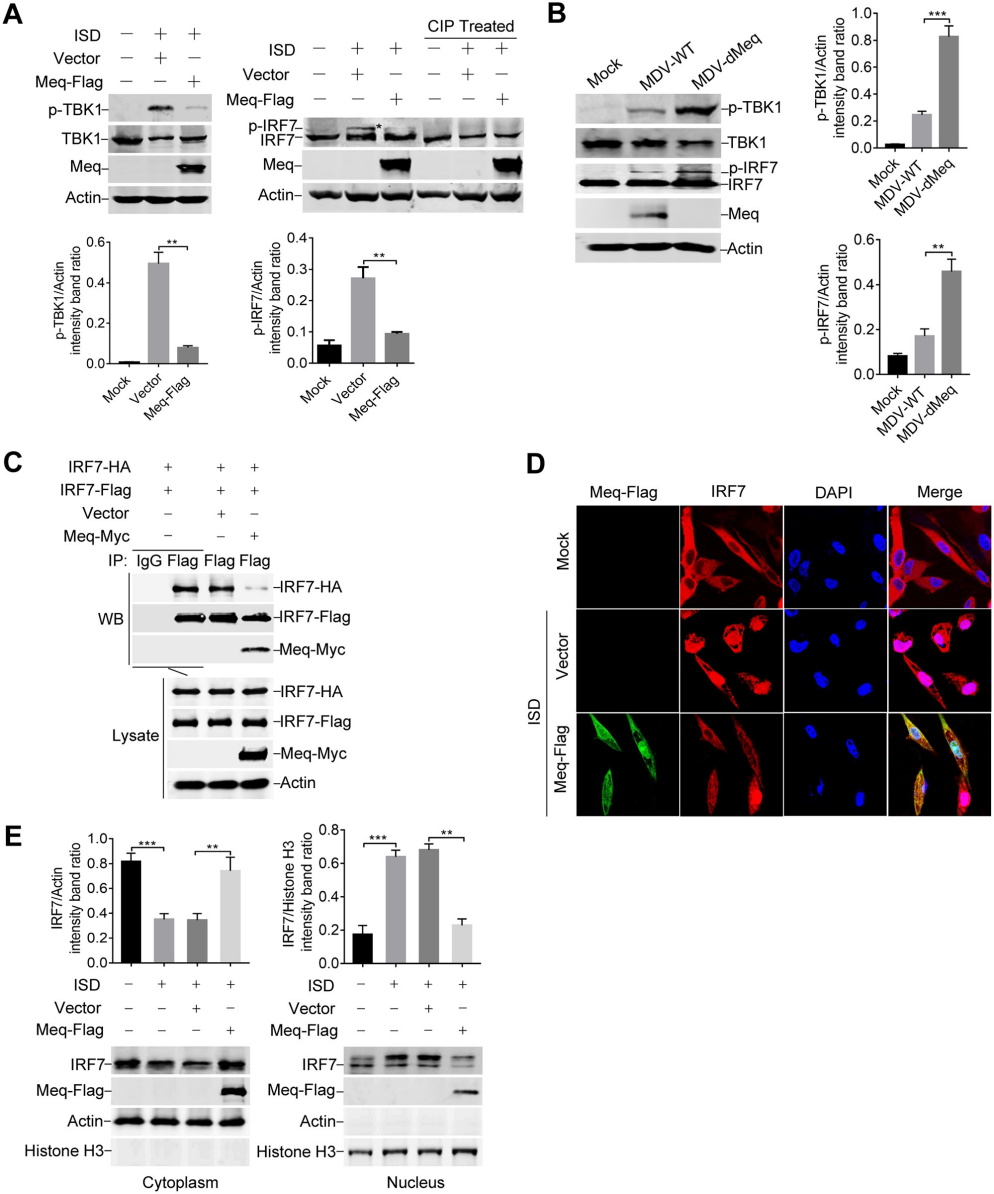
Meq inhibits the phosphorylation and nuclear translocation of IRF7. (A) DF-1 cells were left untreated or transfected with the empty vector or Meq-Flag plasmid and treated with IFN stimulatory DNA (ISD) for 12 h. Cell lysates were left untreated or treated with calf intestine alkaline phosphatase (CIP) for 1 h, and western blotting was performed with the indicated antibodies. The protein levels of phosphorylated TBK1 (p-TBK1) and phosphorylated IRF7 (p-IRF7) were normalized to those of actin; the p-IRF7 protein is indicated by an asterisk (*). (B) CEFs were infected with MDV-WT or MDV-dMeq (MOI = 0.1) for 12 h before immunoblot analysis with the indicated antibodies. (C) DF-1 cells were transfected with the indicated plasmids for 36 h before coimmunoprecipitation and immunoblot analysis with the indicated antibodies. (D) DF-1 cells were transfected with an empty vector or Meq-Flag plasmid, and 24 h later, cells were either left untreated or transfected with ISD for 12 h before confocal microscopy. (E) DF-1 cells were transfected and treated with ISD as indicated and the cell lysates were separated into cytoplasmic and nuclear extracts. The IRF7 protein levels in the cytoplasm and nucleus were analyzed by western blotting. The data represent results from one of the triplicate experiments. **: p < 0.01, ***: p < 0.001.

As phosphorylation of TBK1 and IRF7 is a hallmark of IFN-β induction [6–9], we next examined the effect of Meq on phosphorylation of these proteins. We observed that Meq dramatically inhibited ISD-induced phosphorylation of TBK1 and IRF7 in DF-1 cells (Fig 7A). Consistently, phosphorylation of TBK1 and IRF7 was markedly enhanced in cells infected with the Meq-deficient MDV in comparison to wild-type MDV (Fig 7B). Taken together, these results support the conclusion that, by interacting with STING, Meq impairs assembly of the STING-TBK1-IRF7 complex and prevents the activation of TBK1 and IRF7.

Viral infection triggers IRF7 dimerization and translocation into the nucleus, where it binds to the promoter region to activate IFN-β transcription [32]. As Meq interacted with IRF7 and inhibited its phosphorylation, we next examined whether Meq affects the dimerization and nuclear translocation of IRF7. Coimmunoprecipitation experiments indicated that IRF7 dimerization was markedly decreased in the presence of Meq (Fig 7C). Next, we transfected DF-1 cells with a Meq-Flag expression plasmid or an empty vector, and monitored the ISD-induced nuclear accumulation of IRF7. As shown in Fig 7D, stimulation with ISD increased the IRF7 levels in the nuclei, however, ectopic expression of Meq prevented ISD-stimulated nuclear trafficking of IRF7. We further analyzed the levels of IRF7 in cytoplasmic and nuclear extracts by subcellular fractionation and western blotting, which showed that Meq obviously reduced the level of IRF7 in the nuclei of cells treated with ISD (Fig 7E). These results show that Meq reduced the dimerization and nuclear translocation of IRF7, downstream of its phosphorylation by TBK1.

### Meq plays an important role in MDV immune evasion

**Fig 8:**
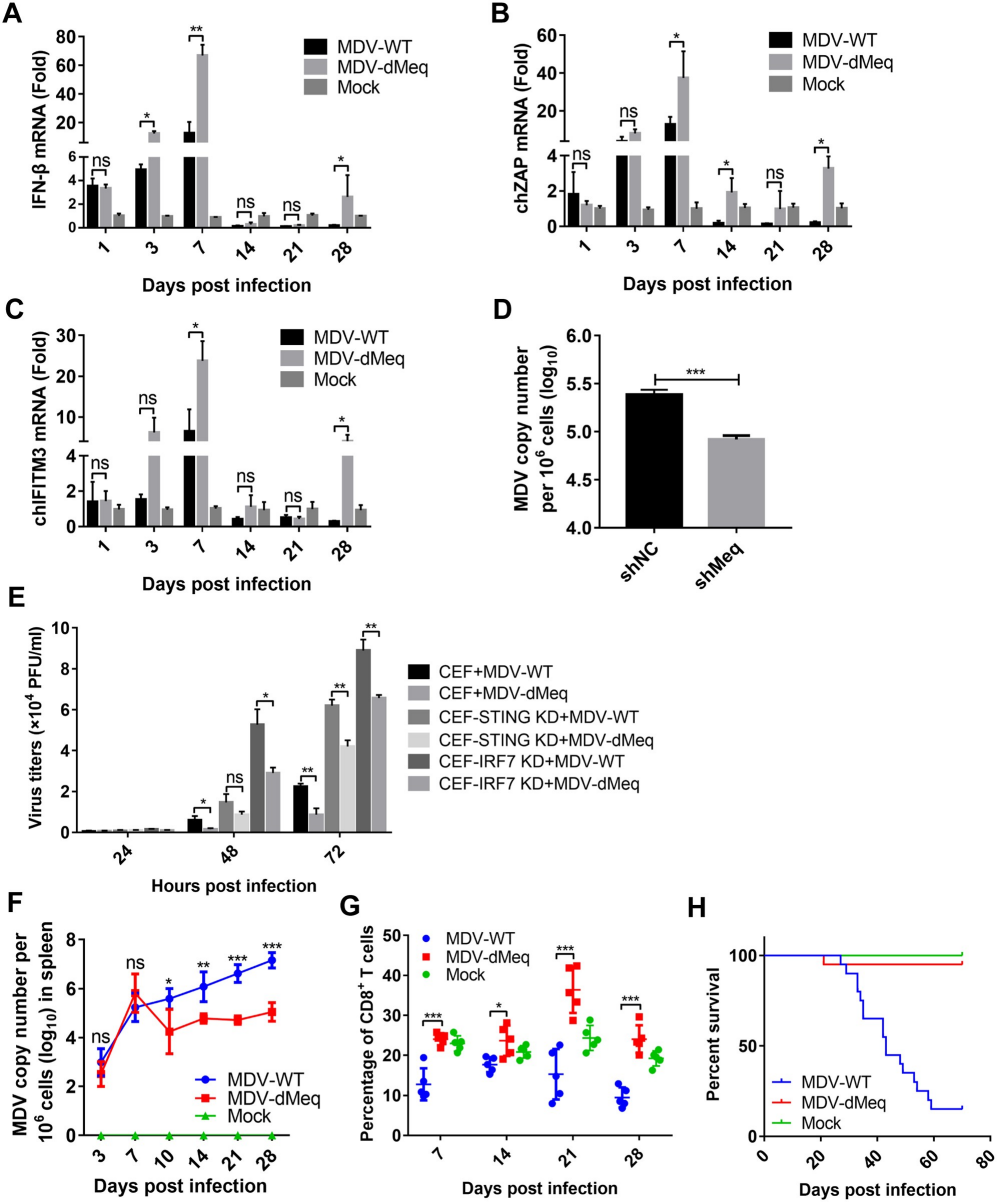
Meq deficiency facilitates IFN-β induction and host CD8+ T cell responses. (A-C) Chickens were infected with 2000 PFUs of wild-type MDV (MDV-WT) or Meq-deficient MDV (MDV-dMeq), and the mRNA levels of *IFN-β* (A), chicken ZAP (ch*ZAP*) (B), and chicken IFN-inducible transmembrane protein 3 (ch*IFITM3*) (C) in the spleen samples were measured by real-time qPCR at the indicated times postinfection. (D) The CEFs transduced with Meq-specific small hairpin RNAs (shMeq) or a control shRNA (shNC) were infected with MDV (MOI = 0.01) for 48 h before the detection of MDV viral titers with real-time qPCR. (E) The indicated cells were infected with wild-type MDV or MDV-dMeq, respectively, and the MDV viral titers were tested using a plaque assay at the indicated time points after infection. (F) One-day-old specific pathogen-free chickens were left untreated or inoculated with MDV-WT or MDV-dMeq, and virus genome copy numbers in the spleen were monitored by real-time qPCR at the indicated time points. (G) Chicken peripheral blood lymphocytes were obtained to analyze the percentage of CD8+ T cells at the indicated time points after infection. (H) The survival rate of chickens after infection. *: p < 0.05, **: p < 0.01, ***: p < 0.001; ns: no significant difference.

To evaluate the role of Meq in the immune evasion of MDV, we infected one-day-old chickens with wild-type or the Meq-deficient MDV, and examined the expression of IFN-β and its downstream antiviral genes. As shown in Fig 8A–8C, the Meq-deficient MDV induced significantly higher levels of *IFN-β*, *ZAP*, and *IFITM3* in chickens than wild-type MDV, especially during the early cytolytic phase (3 and 7 dpi) and the late neoplastic transformation phase (28 dpi).

We next determined the effects of Meq on viral replication in CEFs and in chickens. Knockdown of Meq markedly suppressed viral replication in CEFs (Fig 8D), We further inoculated MDV-WT or MDV-dMeq in wild-type or STING-deficient CEFs. As shown in Fig 8E, both wild-type MDV and the Meq-deficient MDV production in STING-knockdown cells (CEF-STING KD) was increased in comparison to that in control cells, consistent with a critical role of STING in innate antiviral response in chickens. MDV-dMeq produced from wild-type cells decreased approximately 2.58-fold in comparison to wild-type MDV at 72 hpi, whereas MDV-dMeq produced from STING-knockdown cells decreased approximately 1.48-fold in comparison to wild-type MDV (Fig 8E). Since Meq also targeted to IRF7, we constructed IRF7-knockdown CEFs (CEF-IRF7 KD), and found that MDV-dMeq production in IRF7-knockdown cells decreased 1.36-fold in comparison to wild-type MDV at 72 hpi (Fig 8E). These results suggested that Meq promotes the lytic replication of MDV in a cGAS-STING-dependent pathway.

The replication of MDV-WT and MDV-dMeq in vivo was analyzed by qPCR (Fig 8F). It is known that MDV infection in chickens begins with the early cytolytic phase within 3–7 dpi, which is followed by the latency phase between 7 and 10 dpi; MDV reactivation initiates the late cytolytic phase starting around 18 dpi, and finally the transformation phase proceeds with the formation of visceral tumors occurs around 28 dpi [33, 34]. Our *in vivo* experiments showed that the MDV-dMeq virus replicated at the parental MDV-WT level during early cytolytic infection at 3 and 7 dpi. However, the MDV-dMeq viral loads measured beyond 7 dpi were reduced by 10- to 100-fold compared with those of MDV-WT. These results indicated that Meq is dispensable for early cytolytic infection; nonetheless, it may play a role in the subsequent latency, reactivation, and transformation phases.

Recent studies have revealed a link between type I IFNs and CD8+ T cell responses against tumor-associated antigens *in vivo* [35]. Because Meq is able to induce transformation, we suspected that its inhibitory effect on IFN-β induction may affect host antitumor immunity. To test this hypothesis, T cell subsets in the infected chickens were analyzed by flow cytometry. As shown in Fig 8G, the percentage of CD8+ T cells was significantly reduced in chickens infected with MDV-WT compared to those infected with MDV-dMeq. These results suggested that Meq reduces host CD8+ T cell response by inhibiting IFN-β induction during MDV infection in chickens.

In addition, pathogenesis studies indicated that deletion of Meq significantly attenuates the virulence of MDV-dMeq, as only one chicken from the MDV-dMeq group died consequent to nonspecific causes; in comparison, 17 out of 20 chickens in the parental MDV-WT group died during the experiment (Fig 8H). All the chickens in the MDV-WT group exhibited gross MDV-specific lesions, whereas no lesions were observed in the mock- or MDV-dMeq-inoculated groups. These results indicated that the effect of Meq on viral replication and pathogenicity might be due to its ability to inhibit the host immune responses, which resulted in enhanced viral replication and virulence *in vivo*. Taken together, Meq plays an important role in MDV immune evasion and contributes to the replication and oncogenesis of MDV in chickens.

## Discussion

MDV constitutes one of the most contagious and oncogenic herpesviruses [1, 2]. In addition, the virus causes immunosuppression in infected chickens, resulting in increased susceptibility to concurrent or secondary bacterial or viral infections [27]. However, the mechanisms of MDV-induced tumorigenesis and immunosuppression are poorly understood. In the present study, we found that MDV infection in chickens triggered an IFN-β response during the early cytolytic phase, whereas the production of IFN-β and chicken ISGs was inhibited during the reactivation and transformation phases. These observations suggested that MDV is able to modulate host immune responses to evade host surveillance and immunity, which appears to be critical for viral reactivation and transformation during infection. Thus, it was considered worthwhile to determine whether MDV encodes proteins that inhibit IFN-β production along with the underlying mechanisms.

The ability of viruses to evade and modulate the host innate immune response is of central importance for successful establishment and maintenance of infection [5]. The cGAS-STING signaling pathway has been demonstrated to be a key target of herpesviruses for immune evasion [36, 37]. However, in contrast to their mammalian counterparts, avian herpesvirus proteins involved in regulation of this pathway have been rarely studied. In the present study, upon screening over 100 MDV open reading frames (ORFs), we successfully identified a number of viral proteins that counteract the cGAS-STING pathway and inhibit IFN-β induction. Moreover, we found that MDV could escape the host innate immune response by antagonizing the function of STING, a key molecule in the host DNA-sensing pathways [12]. Our results revealed for the first time that the major MDV oncoprotein Meq interacts with STING and IRF7, which prevented the associations of STING-TBK1 and STING-IRF7, leading to the inhibition of IRF7 activation and IFN-β induction. Notably, we showed that overexpression of Meq specifically inhibited DNA virus- and cytosolic dsDNA-induced production of type I IFNs and downstream antiviral genes. Conversely, ablation of Meq triggered a stronger IFN-β response and resulted in attenuated viral replication and transformation. These results suggested that Meq plays a direct role in evasion of the innate antiviral response upon MDV infection.

Given the key role of STING in regulation of the host antiviral response, many viruses have evolved various mechanisms to target this protein for subversion of the host innate immunity [36, 37]. HSV-1 inhibits STING-mediated signaling through the viral proteins UL46 and ICP27 [38, 39]. The KSHV protein vIRF1 and HCMV protein US9 disrupt the STING-TBK1 association through competitive interaction with STING [14, 40]. Another HCMV protein, UL82, impairs the cellular trafficking of STING by disrupting its translocation complex, leading to inhibition of the innate antiviral response and immune evasion by HCMV [41]. The present study adds the MDV oncoprotein Meq to the expanding family of viral proteins that inhibit STING signaling by impairing assembly of the STING-TBK1-IRF7 complex, thereby preventing IRF7 activation and IFN-β induction.

In addition to promote immunity to DNA viruses, it is evident that STING is required for host protection against a number of RNA-related pathogens including vesicular stomatitis virus, Sendai virus, and dengue virus [42–44]. Furthermore, various bacteria have also been reported to promote STING signaling via genomic DNA and secretion of STING-activating cyclic dinucleotides [12, 43]. Therefore, as an inhibitor of STING, Meq might also be able to inhibit the innate immunity against RNA viruses and bacteria. Consistently, our results showed that Meq markedly reduced the IFN-β promoter activity and IFN-β production stimulated by Sendai virus, poly(I:C) and *Escherichia coli* DNA (S3 Fig). In addition, we identified multiple MDV proteins that counteract the cGAS-STING DNA-sensing pathway; these viral proteins might inhibit IFN-β induction by affecting any step in this pathway. The steps downstream of TBK1 or IRF7 activation, for example, are shared by many other pathways, such as the Toll-like receptor and retinoic acid-inducible gene I-like receptor pathways [6, 7]. Thus, these candidates may affect other pathways in addition to the cGAS-STING pathway, leading to the inhibition of IFN-β production triggered by RNA viral and bacterial infection. The findings in our study may explain to some extent why MDV-infected birds exhibit immunosuppression and are more susceptible to concurrent or secondary viral or bacterial infections.

Although Meq is considered the principal viral oncoprotein of MDV, the molecular mechanisms of Meq-induced transformation are not completely understood [19]. Meq protein interactions, as self- or bZIP dimers or with non-bZIP proteins such as C-terminal-binding protein and heat shock protein 70, are reported to be critical for virus oncogenicity [23, 45]. Moreover, Meq is able to antagonize apoptosis of the transformed cells by interacting with p53 and inhibiting its transcriptional and apoptotic activities [24]. Type I IFNs have been implicated in tumor suppression through the induction of tumor cell-specific apoptosis as well as boosting antitumor immunity [46]. cGAS also induces apoptosis through activating STING-TBK1-IRF3 pathway upon DNA sensing during herpesvirus infection [47]. In the present study, we identified Meq as an efficient antagonist of STING signaling, which may also contribute to its anti-apoptotic function.

Besides, the cGAS-STING pathway has been shown to be critical for the innate immune sensing of immunogenic tumors [48–50]. The tumor-derived DNA is recognized by cGAS, which produces cGAMP for STING activation and IFN-β production, facilitating the activation of antitumor CD8+ T cell responses *in vivo* [9, 50]. It was previously reported that priming of CD8+ T cells against tumor-associated antigens is defective in STING-deficient mice [48]. In the present study, we analyzed the T cell subsets in chickens infected with different MDVs. Comparing with those infected with the Meq-deficient virus, chickens infected with the wild-type MDV exhibited significantly reduced CD8+ T cell responses which might be against both the virus and the tumors induced by MDV infection. These results suggested that, by antagonizing STING signaling and inhibiting the IFN-β production triggered by MDV DNA and tumor-derived DNA, Meq suppresses the host innate and adaptive antitumor immune responses, facilitating the establishment of transformation and tumorigenesis. Taken together, this study broadens our understanding of Meq-induced transformation, a process that involves multiple functions of Meq in transactivation, anti-apoptosis, and blocking of the DNA-sensing pathway, which results in the inhibition of type I IFN induction, antitumor immunity, and apoptosis of tumor cells.

**Fig 9:**
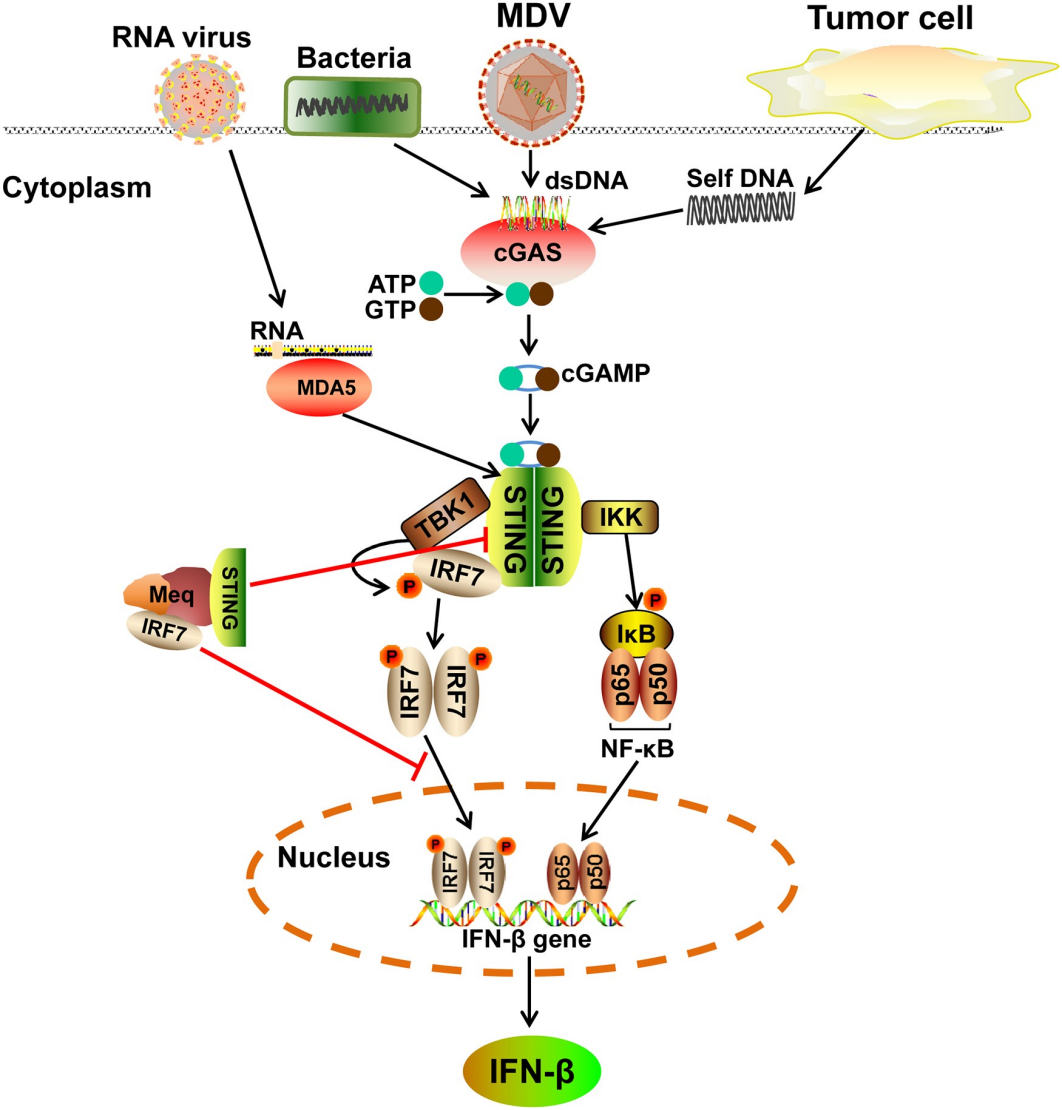
Schematic model of Meq-mediated inhibition of the cGAS-STING pathway during MDV infection. MDV DNA or tumor-derived DNA is recognized by cytosolic DNA sensor cGAS, which produces cGAMP for STING activation and IFN-β production. However, the MDV oncoprotein Meq interacts with STING and IRF7, which disrupts assembly of the STING-TBK1-IRF7 complex, thereby leading to the inhibition of IRF7 activation and IFN-β induction during MDV infection. Additionally, STING is involved in the melanoma differentiation-associated gene 5 (MDA5) signaling pathway against RNA viruses as well as the innate immunity against bacteria. As an inhibitor of the cGAS-STING signaling, Meq could exert its immunomodulatory functions in the innate immune responses against not only DNA viruses but also RNA-related pathogens and various bacteria.

In summary, our findings suggest a new role of the MDV oncoprotein Meq in inhibition of IFN-β production by selective targeting of STING and IRF7 in the DNA-sensing pathway. By directly binding to STING and IRF7, Meq disrupts assembly of the STING-TBK1-IRF7 complex, thereby leading to the inhibition of IRF7 activation and IFN-β induction during viral infection (Fig 9). Given the multiple roles played by the cGAS-STING axis in not only the recognition of a variety of pathogens but also the induction of antitumor immunity and tumor cell-specific apoptosis, the inhibition of cGAS-STING signaling by Meq may contribute to Meq-induced tumorigenesis in addition to establishment of persistent infection. Our findings reveal an important mechanism of immune evasion of MDV, which promotes our understanding of the virus-host interaction in MDV-induced lymphoma and may facilitate the development of more effective vaccines against MDV infection.

## Materials and methods

### Animals and ethics statement

The specific-pathogen-free (SPF) chickens, fertilized SPF chicken eggs and duck eggs used in this study were purchased from State Resource Center of Laboratory Animal for Poultry (Harbin, China). Ten-day-old SPF chicken embryos were used for preparation of primary CEFs and 12-day-old SPF duck embryos were used for preparation of primary duck embryo fibroblasts (DEFs). This study was carried out in strict accordance with the recommendations in the Guide for the Care and Use of Laboratory Animals of the Ministry of Science and Technology of China [51]. The use of SPF eggs, embryos and chickens and the animal experiments were approved by the Animal Ethics Committee of Harbin Veterinary Research Institute of the Chinese Academy of Agricultural Sciences and performed in accordance with animal ethics guidelines and approved protocols (SYXK (Hei) 2017–009).

### Cells, viruses, and antibodies

DF-1 (ATCC CRL-12203) and HEK293T (ATCC CRL-3216) cells were cultured in Dulbecco’s Modified Eagle’s Medium (DMEM, Life Technologies, Grand Island, NY) containing 10% fetal bovine serum (FBS, Sigma-Aldrich, St. Louis, MO). Primary CEFs were prepared from 10-day-old SPF chicken embryos and primary DEFs were prepared from 12-day-old SPF duck embryos. CEFs and DEFs were cultured in DMEM supplemented with 10% FBS (Sigma-Aldrich, St. Louis, MO). The virulent MDV GA strain (GenBank no. AF147806) and HVT FC126 strain (GenBank no. AF291866) were propagated in CEFs or DF-1 cells prior to use in this study. Antibodies including mouse anti-Flag, rabbit anti-hemagglutinin (HA), mouse anti-c-Myc, rabbit anti-c-Myc, mouse anti-actin (Sigma-Aldrich, St. Louis, MO, USA), rabbit anti-TBK1, and rabbit anti-phospho-TBK1 (Cell Signaling Technology, Boston, MA, USA) were used, along with rabbit anti-STING, rabbit anti-IRF7, mouse anti-Meq, and mouse anti-gI antibodies, which were prepared in our laboratory. ISD, poly(dA:dT) and poly(I:C) were purchased from InvivoGen (San Diego, CA, USA).

### Plasmid constructs

The MDV ORFs were amplified from the genome of the virulent MDV GA strain and cloned into the pCAGGS vector with a Flag tag fused to the 3′ ends. Plasmids encoding chicken cGAS (GenBank no. XM_419881), STING (GenBank no. KP893157), TBK1 (GenBank no. NM_001199558), IRF7 (GenBank no. KP096419), IKKβ (GenBank no. NM_001031397), and MDA5 (GenBank no. AB371640.1) were constructed by cloning the synthesized sequence into pCAGGS with a Flag or HA tag fused to the 3′ end. The chicken *IFN-β* promoter luciferase reporter pchIFN-β-luc was constructed by inserting the −158 to +14 fragment of the chicken *IFN-β* promoter into the pGL3-basic vector, as described previously [29, 52]. The pIRF7-luc reporter contained four copies of the IRF7-binding positive regulatory domain (GCA AAT AGA AAG C), and the pNF-κB-luc reporter contained four copies of the NF-κB-binding positive regulatory domain (GGG AAT TCT C).

### Real-time qPCR

Total RNA was extracted from cells using the RNAiso Plus reagent (TaKaRa, Otsu, Japan). Reverse transcription was performed using the ReverTra Ace qPCR RT Kit (Toyobo, Osaka, Japan). The quantity of each cDNA was determined by real-time qPCR using Thunderbird SYBR qPCR mix (Lucigen, Madison, WI, USA) and analyzed with the LightCycler 480 system (Roche, Basel, Switzerland). Specific primers for *IFN-β*, chicken *ZAP* (*chZAP)*, and chicken *IFITM3* (*chIFITM3*) were synthesized by Invitrogen (Shanghai, China), and the relative mRNA levels of these genes were normalized to the chicken *β-actin* mRNA level in each sample. The fold differences between the treated samples and mock samples were calculated. To determine the MDV viral titers, total DNA was extracted using the AxyPrep BodyFluid Viral DNA/RNA Miniprep Kit (Corning Life Sciences, Shanghai, China) and tested with real-time qPCR by measuring the copy numbers of the MDV *Meq* gene as an MDV genome target (the sequences amplified by the *Meq* primers remained in the genome of MDV-dMeq) and the chicken ovotransferrin gene as a reference, as described previously [53]. All controls and treated samples were examined in triplicate in the same plate.

### ELISA

The IFN-β protein levels in cell cultures were analyzed using a chicken IFN-β ELISA kit (USCN Life Science, Wuhan, China) according to the manufacturer’s instructions.

### Transfection and dual-luciferase reporter assays

To determine chicken *IFN-β* promoter, IRF7, and NF-κB binding activities, DF-1 cells seeded in 24-well plates were cotransfected with a firefly luciferase reporter plasmid (IFN-β-luc, IRF7-luc, or NF-κB-luc) and *Renilla* luciferase reporter pRL-TK, which served as an internal control, with or without expression plasmids, as indicated, using the TransIT-X2 dynamic delivery system (Mirus, Madison, WI, USA). At 36 h posttransfection, cells were lysed, and samples were assayed for firefly and *Renilla* luciferase activity using the dual-luciferase reporter assay system (Promega, Madison, WI, USA). Relative luciferase activity was normalized to *Renilla* luciferase activity. The reporter assays were repeated at least three times.

### Construction of Meq-expressing cells

The Meq-encoding sequence was cloned into the pLVX-IRES-ZsGreen1 lentiviral vector (Clontech, Mountain View, CA, USA) with a Flag tag fused to its 3' end. The recombinant plasmid pLVX-Meq was sequenced and packaged in HEK293T cells with the helper plasmids psPAX2 and pMD2.G. The resulting lentiviral expression plasmid was transduced into DF-1 cells, and stably transduced cells were selected by flow cytometry. The expression of Meq was detected by western blotting.

### Knockdown of Meq by shRNA lentiviral interference

A lentiviral vector-based siRNA plasmid (piLenti-shMeq-GFP) expressing shRNA that targets Meq was designed and constructed by Applied Biological Materials (Richmond, BC, Canada). The piLenti-shMeq-GFP plasmid was transduced into CEFs according to the manufacturer’s instructions to establish stable Meq knockdown cells. CEFs transduced with the same vector plasmid expressing a scrambled shRNA served as a negative control. The stably transduced cells were monitored using green fluorescent protein (GFP) and selected by flow cytometry. The knockdown efficiency of Meq was detected by real-time qPCR and western blotting.

### Generation of Meq-deleted recombinant MDV

In our preliminary studies, six fosmid clones, GA1 to GA6, containing sequences encompassing the entire genome of the virulent MDV GA strain were constructed and used for the generation of MDV mutant lacking the *Meq* gene (Fig 4F). Fosmids GA1 and GA5, containing a copy of the coding sequence of Meq, were used for the deletion of this gene with the Counter-Selection BAC Modification Kit (Gene Bridges, Heidelberg, Germany). The GA1 and GA5 fosmid clones in which the *Meq* gene was deleted, designated GA1dMeq and GA5dMeq, were identified by PCR analyses and sequencing. To rescue Meq-deleted recombinant virus, MDV-dMeq, 2 μg of each NotI-digested and purified fosmid DNA (GA1dMeq, GA2, GA3, GA4, GA5dMeq, and GA6) was used to transfect primary DEFs in 60-mm dishes using the calcium phosphate procedure [54]. Five days after transfection, cells were trypsinized, seeded onto a 100-mm dish, and monitored for cytopathic effects. Viral stocks were subsequently generated in DEFs for further analysis.

### Coimmunoprecipitation assays and western blot analysis

The expression plasmids harboring Flag or HA tags were transfected into HEK293T or DF-1 cells using the TransIT-X2 dynamic delivery system (Mirus). At 36 h posttransfection, cells were lysed in ice-cold Pierce IP buffer (Thermo Fisher Scientific, Waltham, MA, USA) containing protease inhibitor cocktail (Roche). The lysates were obtained by centrifugation and incubated with the indicated antibodies at 4 °C overnight. Protein G Sepharose beads (Roche) were added, and samples were incubated for another 6 h. The beads were washed six times with phosphate-buffered saline and boiled in sodium dodecyl sulfate loading buffer before analysis by western blotting with the indicated antibodies.

For western blotting, whole-cell lysates were obtained by lysing cells in NP-40 lysis buffer (Beyotime, Beijing, China). The cytoplasmic and nuclear proteins were extracted using NE-PER nuclear and cytoplasmic extraction reagents (Thermo Fisher Scientific). Protein concentrations were determined with a bicinchoninic acid protein assay kit (Thermo Fisher Scientific). The proteins were separated by electrophoresis on 12% SDS-polyacrylamide gels, transferred onto nitrocellulose membranes, and incubated with the indicated primary and secondary antibodies. Images were acquired with the Odyssey infrared imaging system (LI-COR Biosciences, Lincoln, NE, USA).

### GST pull-down assay

GST-STING or GST-IRF7 was bound to glutathione agarose beads, and incubated for 4 hours with lysates from HEK293T cells transiently expressing Meq-Flag at 4°C. The beads were washed five times each with NP-40 lysis buffer (Beyotime Biotechnology, Shanghai, China), mixed with 5× SDS-loading buffer and boiled for 10 min. The input/elutes were resolved by SDS-PAGE and analyzed by Coomassie staining and/or immunoblot analysis.

### Confocal imaging

DF-1 cells were transfected with the plasmids using the TransIT-X2 dynamic delivery system, and 24 h later, they were treated with ISD for another 12 h. For confocal imaging, cells were firstly fixed with 4% paraformaldehyde for 30 min and permeabilized with 0.1% Triton X-100 in PBS for 15 min, which was followed by blocking with 5% bovine serum albumin in PBS for 1 h. Then, the cells were incubated with rabbit anti-IRF7 and mouse anti-Flag antibodies for 1 h. The cells were washed five times with PBS and incubated with the Alexa 546-anti-rabbit and Alexa 488-anti-mouse secondary antibodies (Abcam). Finally, nuclei were stained with 4′,6-diamidino-2-phenylindole (DAPI; Sigma-Aldrich). After washing five times with PBS, the cells were examined using a confocal microscope system (Zeiss LSM880, Oberkochen, Germany).

### RNA interference

siRNAs specifically targeting chicken STING (5’-AGG TGC TGT GTT CCT GCT TCC-3’) and IRF7 (5’-GGA GCA CTC ACA TGT TCA TGC-3’) as well as a scramble negative control siRNA (5’-GTT CTC CGA ACG TGT CAC GT-3’) were synthesized by GenePharma (Shanghai, China). The siRNA transfections were performed in CEFs using TransIT-X2 dynamic delivery system (Mirus) according to the manufacturer’s instructions. Twenty-four hours after transfection, cells were harvested or infected with MDV for further analysis. The knockdown efficiency of STING or IRF7 was verified by real-time qPCR and western blotting.

### Animal studies

To determine the effects of MDV infection on the induction of *IFN-β* and downstream antiviral genes, 45 one-day-old specific pathogen-free chickens were inoculated subcutaneously on the back of the neck with 2000 PFUs of the virulent MDV GA strain, and the mock control group containing 45 chickens was inoculated with DMEM. At the indicated time points as shown in Fig 1D–1F, spleen samples were collected from five birds in each group, and the mRNA levels of *IFN-β* and chicken ISGs were measured by real-time qPCR.

To characterize MDV-WT and MDV-dMeq viruses, a total of 105 one-day-old specific pathogen-free chickens were randomly divided into three groups, with 35 chickens in each group. Two groups were inoculated subcutaneously with 2000 PFUs of MDV-WT or MDV-dMeq, and the third group was mock-injected with DMEM. On days 1, 3, 7, 10, 14, 21, and 28, five birds from each group were humanely euthanized by electronarcosis and cervical dislocation. Spleen samples were collected for analysis of IFN-β and chicken ISG expression and viral DNA copy numbers, and anticoagulated blood samples were collected to obtain peripheral blood lymphocytes using a chicken peripheral blood lymphocyte separation fluid kit (TBD, Tianjin, China). The cell suspensions were stained with fluorescein isothiocyanate (FITC)-conjugated anti-chicken CD4, R-phycoerythrin-conjugated anti-chicken CD8a, and R-phycoerythrin/Cyanine 5 (SPRD)-conjugated anti-chicken CD3 monoclonal antibodies (SouthernBiotech, Birmingham, AL, USA) for 30 min at 4 °C. After washing with phosphate-buffered saline, the relative immunofluorescence of cells was analyzed using a flow cytometer (Cytomics TM FC 500, Beckman Coulter, Brea, CA, USA).

### Statistical analysis

All experiments were performed at least three times unless otherwise indicated; data are presented as the means ± standard deviations (SD). Statistical significance between groups was determined by Student’s *t* test with GraphPad Prism 7.0 software (La Jolla, CA, USA). A p value of <0.05 was considered statistically significant.

## Supporting information

S1 Fig Effects of the top five MDV open reading frames (ORFs) on TBK1- and IRF7-mediated IFN-β promoter activation. The top five MDV ORF inhibitors and the gI ORF were cotransfected with TBK1 (A) or IRF7 (B) expression plasmids and the IFN-β-luc reporter into DF-1 cells. The dual-luciferase reporter assay was performed 36 h posttransfection, and the fold relative to the mock controls was determined. ***: p < 0.001; ns: no significant difference.
(TIF)

S2 Fig Meq does not affect the associations of STING-IKKβ and STING-MDA5. DF-1 cells were cotransfected with STING-Flag and IKKβ-HA (A) or MDA5-HA (B) with or without Meq-Myc for 36 h before coimmunoprecipitation and immunoblot analysis with the indicated antibodies.
(TIF)

S3 Fig Meq inhibits the IFN-β promoter activation and IFN-β transcription induced by Sendai virus (SeV), poly(I:C) and *Escherichia coli* DNA. (A) DF-1 cells were cotransfected with IFN-β-luc reporter plasmid along with pRL-TK control plasmid and empty vector or the Meq expression plasmid, and 24 h after transfection, cells were infected with SeV or transfected with poly(I:C) and *E*. *coli* DNA as indicated. The luciferase activity was measured 16 h later, and fold activation was determined relative to that for empty vector with mock treatment. (B) DF-1 cells were transfected with empty vector or the Meq expression plasmid, and 24 h after transfection, cells were infected with SeV or transfected with poly(I:C) and *E*. *coli* DNA as indicated. The *IFN-β* mRNA was measured by real-time qPCR 12 h later, and fold relative to that for empty vector with mock treatment was determined. **: p < 0.01, ***: p < 0.001; ns: no significant difference.
(TIF)
