# Supplementary figures and images for: Avian oncogenic herpesvirus antagonizes the cGAS-STING DNA-sensing pathway to mediate immune evasion
Source: PLoS Pathog. 2019 Sep 20;15(9):e1007999. doi: 10.1371/journal.ppat.1007999 (PMC6799934; doi:10.1371/journal.ppat.1007999)

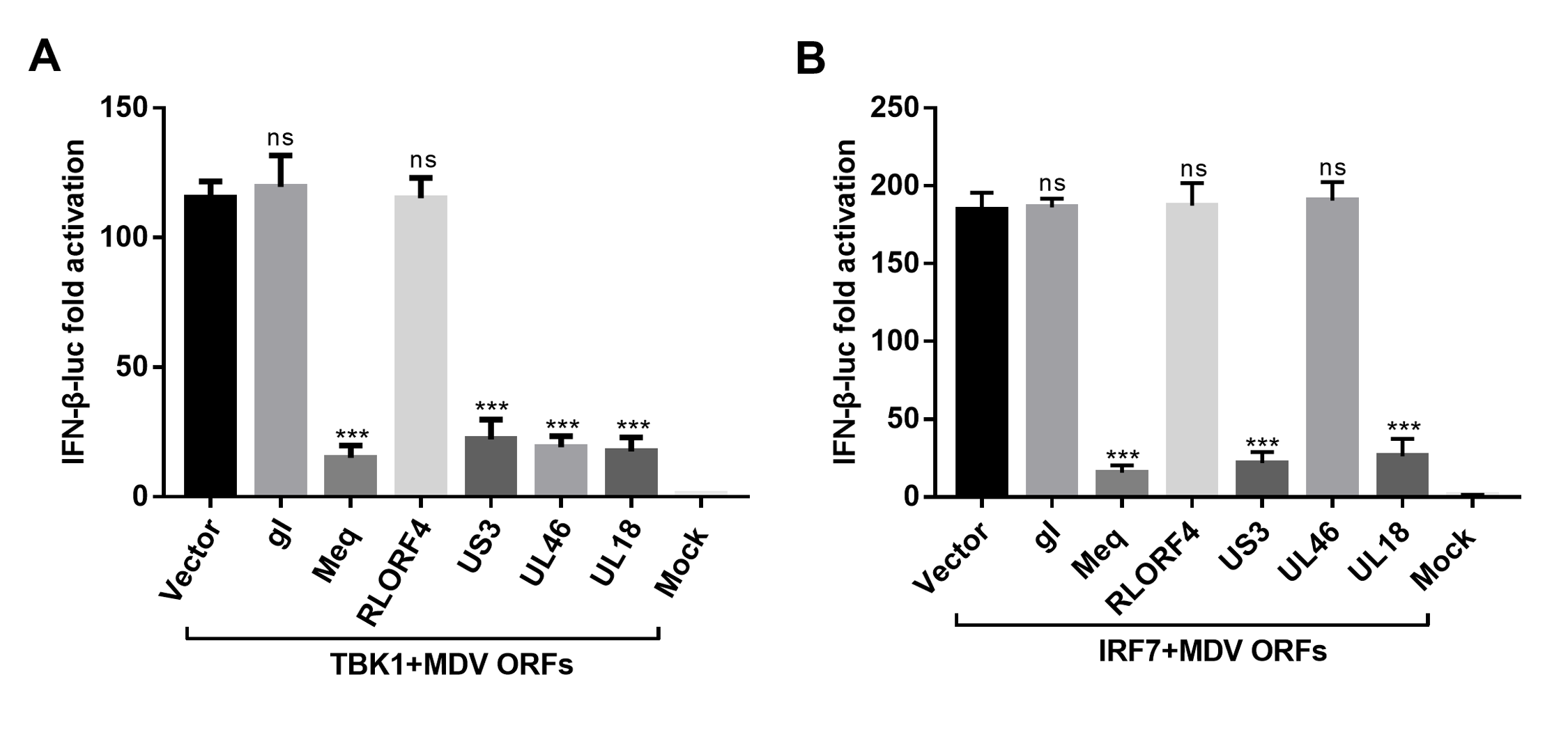

Supplement: S1 Fig — The top five MDV ORF inhibitors and the gI ORF were cotransfected with TBK1 (A) or IRF7 (B) expression plasmids and the IFN-β-luc reporter into DF-1 cells. The dual-luciferase reporter assay was performed 36 h posttransfection, and the fold relative to the mock controls was determined. ***: p < 0.001; ns: no significant difference. (TIF) [file ppat.1007999.s001.tif]

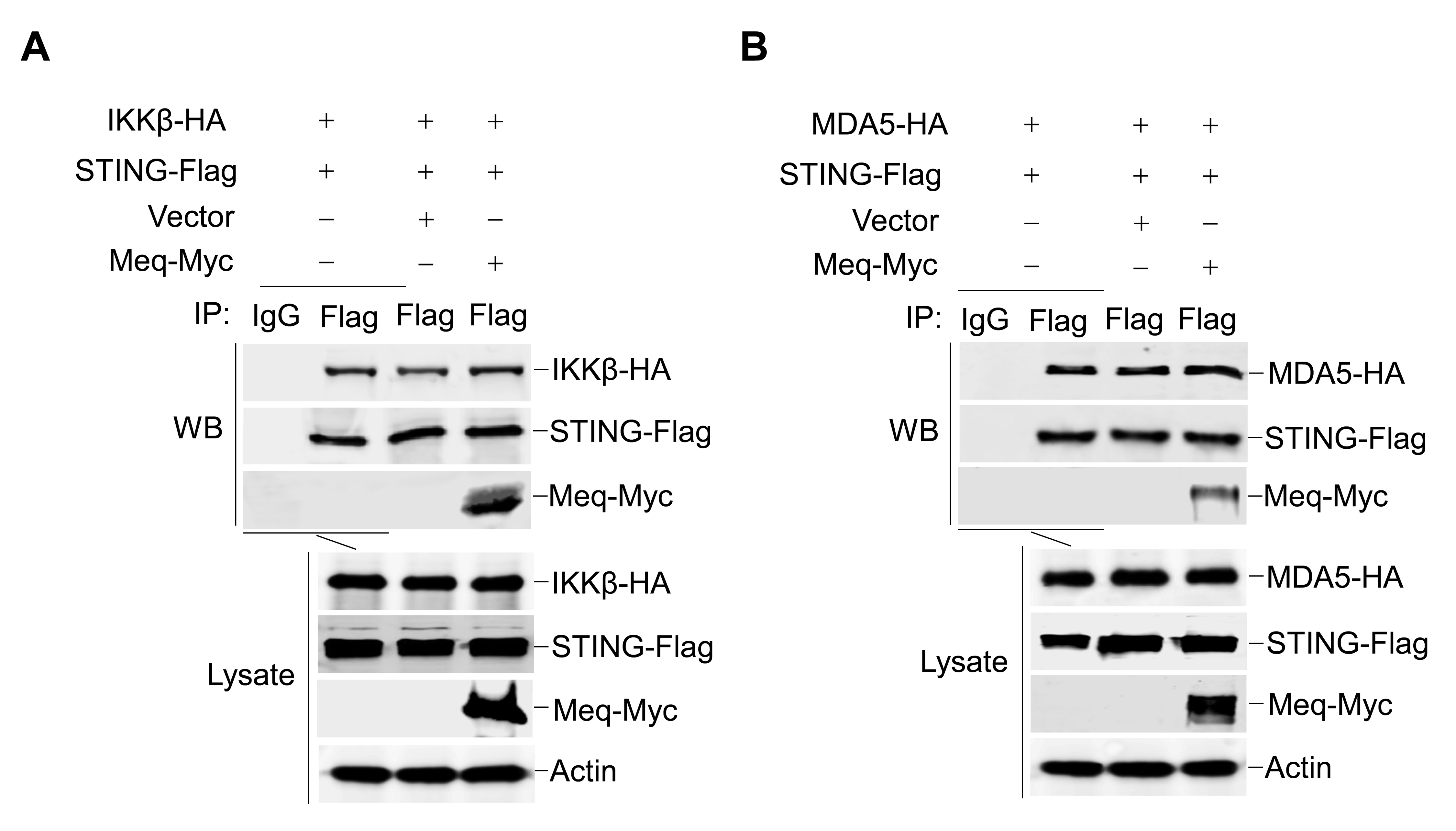

Supplement: S2 Fig — DF-1 cells were cotransfected with STING-Flag and IKKβ-HA (A) or MDA5-HA (B) with or without Meq-Myc for 36 h before coimmunoprecipitation and immunoblot analysis with the indicated antibodies. (TIF) [file ppat.1007999.s002.tif]

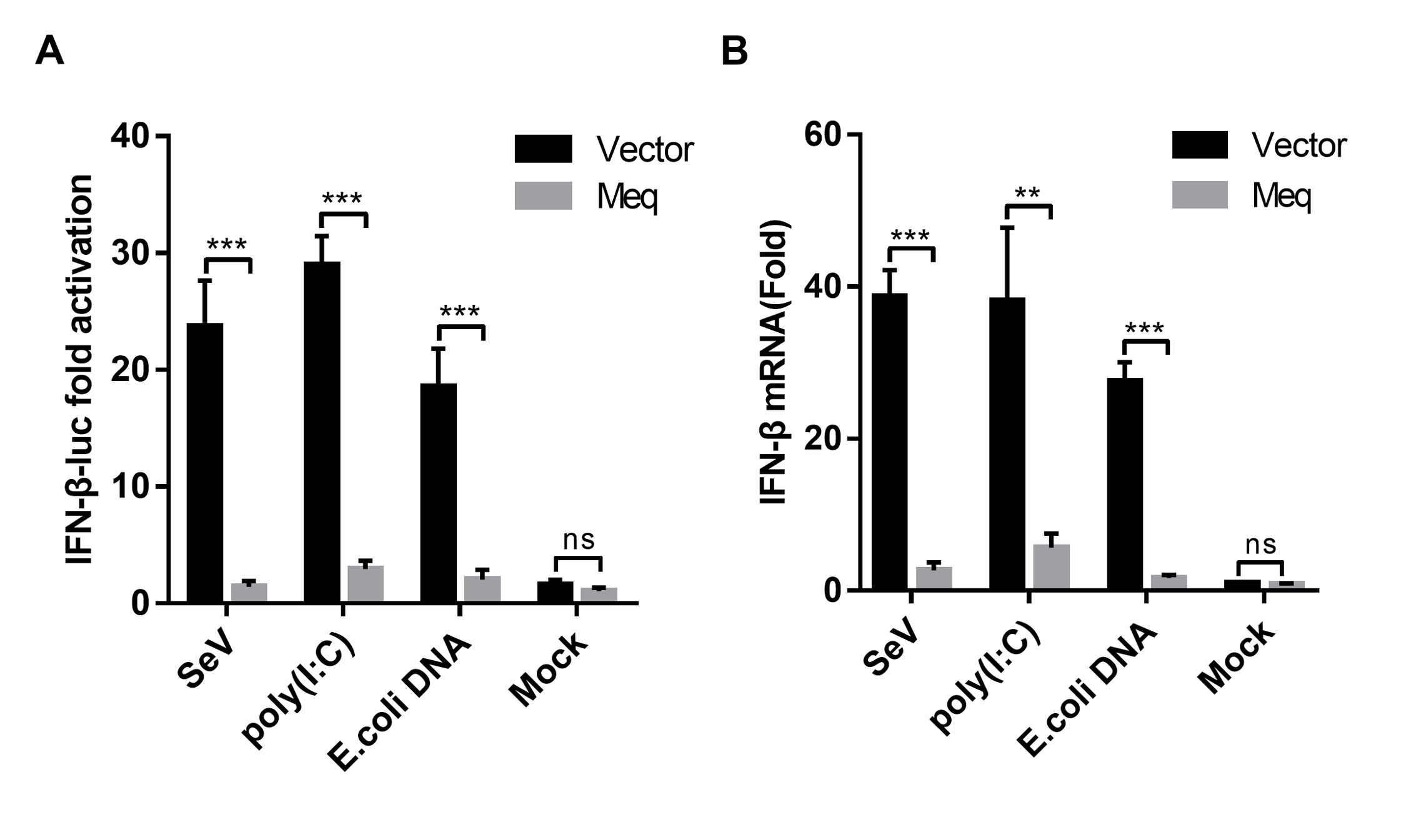

Supplement: S3 Fig — (A) DF-1 cells were cotransfected with IFN-β-luc reporter plasmid along with pRL-TK control plasmid and empty vector or the Meq expression plasmid, and 24 h after transfection, cells were infected with SeV or transfected with poly(I:C) and E. coli DNA as indicated. The luciferase activity was measured 16 h later, and fold activation was determined relative to that for empty vector with mock treatment. (B) DF-1 cells were transfected with empty vector or the Meq expression plasmid, and 24 h after transfection, cells were infected with SeV or transfected with poly(I:C) and E. coli DNA as indicated. The IFN-β mRNA was measured by real-time qPCR 12 h later, and fold relative to that for empty vector with mock treatment was determined. **: p < 0.01, ***: p < 0.001; ns: no significant difference. (TIF) [file ppat.1007999.s003.tif]
